# P2X4 receptor–eNOS signaling pathway in cardiac myocytes as a novel protective mechanism in heart failure

**DOI:** 10.1016/j.csbj.2014.11.002

**Published:** 2014-11-07

**Authors:** Ronghua Yang, Dardan Beqiri, Jian-Bing Shen, John M. Redden, Kimberly Dodge-Kafka, Kenneth A. Jacobson, Bruce T. Liang

**Affiliations:** a Pat and Jim Calhoun Cardiology Center, University of Connecticut Medical Center, Farmington, CT, United States; b Laboratory of Bioorganic Chemistry, NIDDK, National Institutes of Health, Bethesda, MD, United States

**Keywords:** Cardiac myocyte, Cardioprotection, Purines, Heart failure

## Abstract

We have demonstrated using immunoprecipitation and immunostaining a novel physical association of the P2X4 receptor (P2X4R), a ligand-gated ion channel, with the cardioprotective, calcium-dependent enzyme endothelial nitric oxide synthase (eNOS). Treatment of murine ventricular myocytes with the P2XR agonist 2-methylthioATP (2-meSATP) to induce a current (mainly Na^+^) increased the formation of nitric oxide (NO), as measured using a fluorescent probe. Possible candidates for downstream effectors mediating eNOS activity include cyclic GMP and PKG or cellular protein nitrosylation. A cardiac-specific P2X4R overexpressing mouse line was protected from heart failure (HF) with improved cardiac function and survival in post-infarct, pressure overload, and calsequestrin (CSQ) overexpression models of HF. Although the role of the P2X4R in other tissues such as the endothelium and monocytes awaits characterization in tissue-specific KO, cardiac-specific activation of eNOS may be more cardioprotective than an increased activity of global systemic eNOS. The intra-myocyte formation of NO may be more advantageous over NO derived externally from a donor. A small molecule drug stimulating this sarcolemmal pathway or gene therapy-mediated overexpression of the P2X4R in cardiac myocytes may represent a new therapy for both ischemic and pressure overloaded HF.

## 1 Introduction

Since the original proposal of purinergic transmission by Burnstock [1], the field of purinergic receptors and signaling has grown exponentially. Extracellular ATP and adenosine activating their P2 and P1 purinergic receptors, respectively, mediate a growing number of biological functions. Adenosine (P1) and P2Y_12_ receptors have already become targets for cardiovascular disease [2], [3]. P2X receptors (P2XRs) are trimeric ion channels with a molecular weight of each monomer of 43,438 Da (mouse) or 43,369 Da (human). This receptor channel is activated by ATP and its analogues. The subunit composition of a given P2X channel may be homotrimeric, i.e. of the same P2XR protein, or heterotrimeric, which is known to affect the ligand pharmacology. In the current review, we summarized a novel feature of the P2X4R in that the receptor is not just an ion channel, but it also physically associates with the enzyme endothelial nitric oxide synthase (eNOS). In the cardiac myocyte, we show that P2X4R stimulation causes activation of eNOS, as demonstrated by increased nitric oxide (NO) formation, with two structurally different NO-sensitive fluorescent dyes. Functionally, the P2X4R–eNOS pathway is important in mediating cardioprotection in heart failure (HF).

## 2 Identification and characterization of cardiac myocyte P2X4 receptors

Adult ventricular myocytes from mice, rat and guinea pig show an inward current in response to extracellular ATP [3]. ATP elicited an increase in a non-selective cation current with a reversal potential near 0 mV, which is similar to that of cloned P2X4Rs [4]. In murine ventricular myocytes, the current induced by the P2XR agonist 2-methylthioATP (2-meSATP) is partially insensitive to antagonism by suramin and pyridoxalphosphate-6-azophenyl-2′,4′-disulphonic acid (PPADS), which is also characteristic of the P2X4R [4]. As an ion channel, the P2X4R conducts current carried by both Na^+^ and Ca^2 +^. Although the P2X4R is a calcium permeant channel, the majority of the inward current is carried by Na^+^. Initially, it was predicted that this channel would be deleterious in the heart because of the potential calcium overload caused by the influx of Na^+^ and Ca^2 +^. Under basal control conditions, mice with cardiac-specific P2X4R overexpression were overtly normal without cardiac hypertrophy or failure as they age [5]. Paradoxically, these mice are protected from HF with improved cardiac function and survival with post-infarct, pressure overload, and calsequestrin (CSQ) overexpression-induced HF [6], [7], [8].

## 3 Regulation of P2X receptors in diseased hearts

A question arises as to whether cardiac P2X receptors are regulated during pathological conditions. For example, are these receptors up- or down-regulated in disease states such as heart failure? As we gain understanding of the role of cardiac P2X receptors, their regulation in diseased hearts may have implications for the progression of the disease. In our previous study, the expression of the cardiac P2X4 receptor is increased in the CSQ mice-overexpressing model of hypertrophy and dilated cardiomyopathy. The P2X agonist-stimulated current is also greater in ventricular myocytes isolated from the CSQ mice compared to WT mice [8], consistent with a potential pathophysiological significance of the cardiac P2X4 receptor. In another study by Musa et al. [9], P2X4 mRNA was up-regulated in the sinoatrial node in rats with coronary artery ligation-induced heart failure, with a trend toward an increased P2X4 receptor expression in the left ventricle and right atrium as well. P2X4 receptor expression was also shown to be up-regulated in right ventricles of rats exposed to hypoxia [10]. In post-infarct WT hearts, the P2X4 receptor expression is not changed in the remote non-infarcted region as assessed by immunoblotting (unpublished observation). Studies on human hearts also suggest that all of the P2X subtypes are detectable in the myocardium [11], with P2X4 receptors expressed in failing hearts at the same level as in healthy donor hearts.

## 4 P2X4R associates with eNOS and can induce NO formation with downstream signaling

**Fig. 1:**
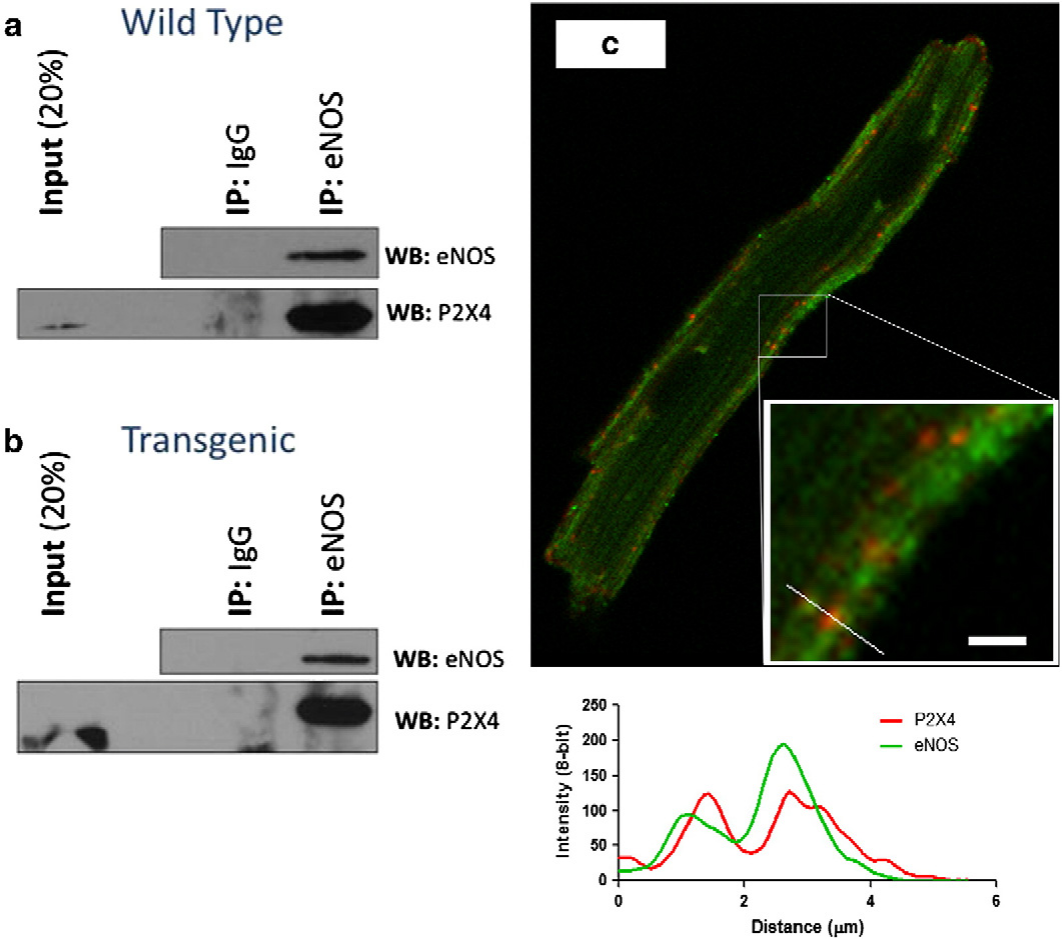
P2X4R and eNOS physically associate with each other as evidenced by co-immunoprecipitation and co-localization in cardiac ventricular myocytes of WT and P2X4R Tg mice. (a) WT myocyte lysates were incubated with anti-eNOS antibody or with non-specific IgG as control. The isolated complex was probed with eNOS (top panel) and P2X4 (bottom panel) antibodies using western blotting. In a control experiment, eNOS co-immunoprecipitated itself. Co-immunoprecipitation of P2X4R with eNOS antibody (lane 3) but not with control IgG (lane 2) was shown. (b) Same experiment as in (a) conducted in P2X4-Tg myocytes. (c) Immunostaining of eNOS (green), P2X4R (red), and merged image was shown for a P2X4R-overexpressing Tg cardiac myocyte.

Since the P2X4R is calcium permeant, it is possible that this receptor can interact with and activate the calcium-dependent endothelial nitric oxide synthase (eNOS). Data summarized in Fig. 1 show that P2X4R is co-localized with eNOS in the sarcolemma (Fig. 1c) and is present in eNOS immunoprecipitates obtained from cardiac myocytes of both WT (Fig. 1a) and P2X4R Tg (Fig. 1b) hearts. The specificity of this physical interaction is supported by the absence of P2X4R in immunocomplexes captured using an IgG matched control antibody.

In the immunostaining, the two proteins co-localized over a specific region in the myocyte, as demonstrated by plotting staining over distance (Fig. 1c inset with the line plot). Both proteins co-localized in the merged staining. Both proteins also showed sarcolemmal staining and co-localization in WT myocytes, although P2X4R staining was considerably fainter [12], likely reflecting the low level of endogenous receptor expression. Despite this, we observed the co-localization of P2X4R with eNOS at the sarcolemma of WT cardiac myocytes.

**Fig. 2:**
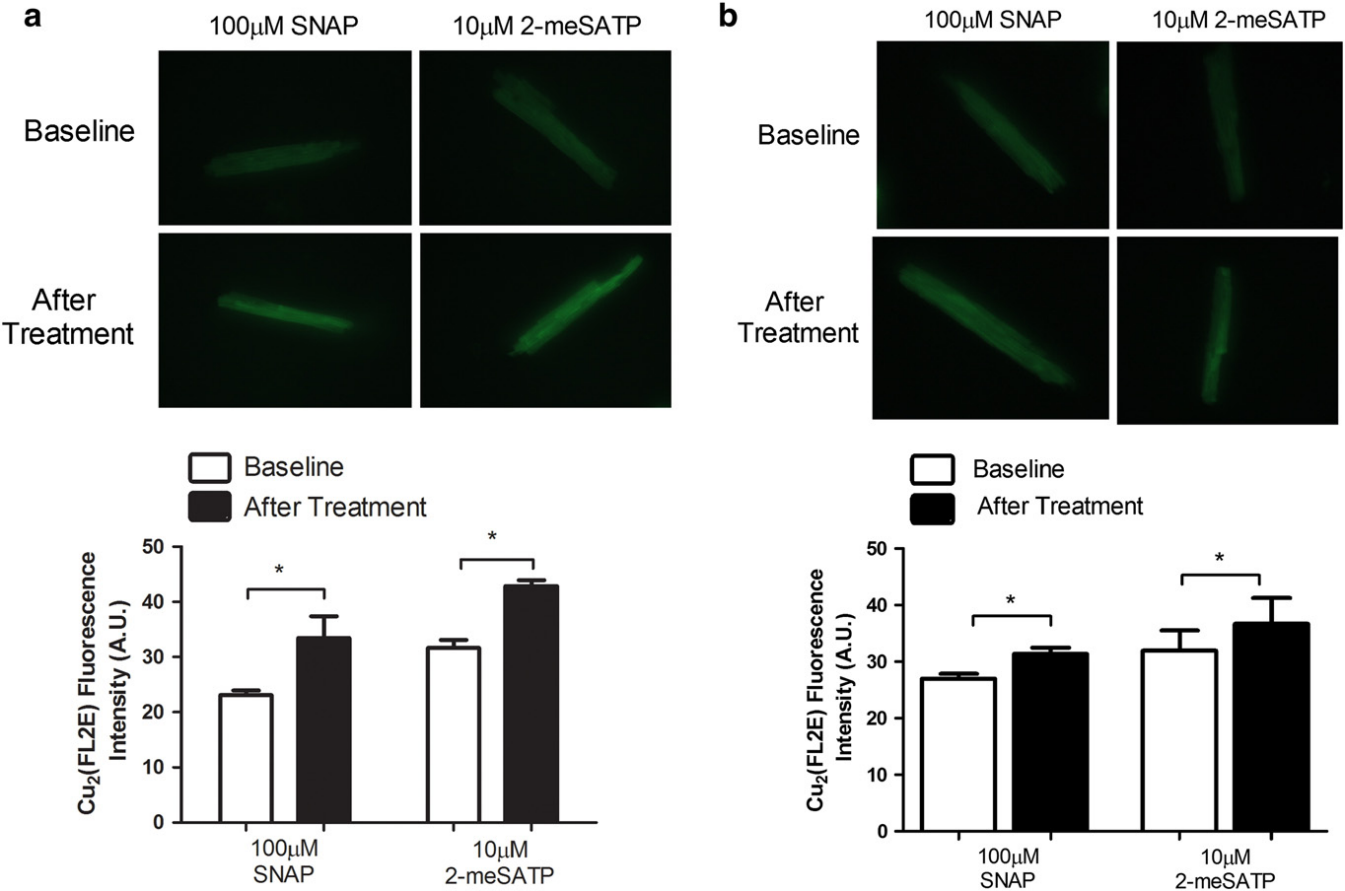
P2X agonist 2-meSATP stimulated cardiac myocyte NO formation in P2X4R Tg and WT animals. Upper panel: Representative fluorescence images of Cu_2_(FL2E) loaded P2X4R-overexpressing Tg cardiac ventricular myocytes before and after SNAP or 2-meSATP treatment are shown (a). Lower figure: *P < 0.05 using paired t-test for average fluorescence intensity in arbitrary units (A.U.) before and after treatment with SNAP (4 = mice) or 2-meSATP (n = 3 mice) from P2X4R Tg animals (a). (b) Similar studies were carried out in cardiac ventricular myocytes from WT animals (n = 3 mice). Upper and lower panels show typical fluorescence imaging and average A.U. for treatment with SNAP and with 2-meSATP. *P < 0.05 using paired t-test for average fluorescence intensity in arbitrary units (A.U.) before and after treatment with SNAP or 2-meSATP. In myocytes isolated from each mouse of WT or Tg animals, 20–80 cells were counted.

**Fig. 3:**
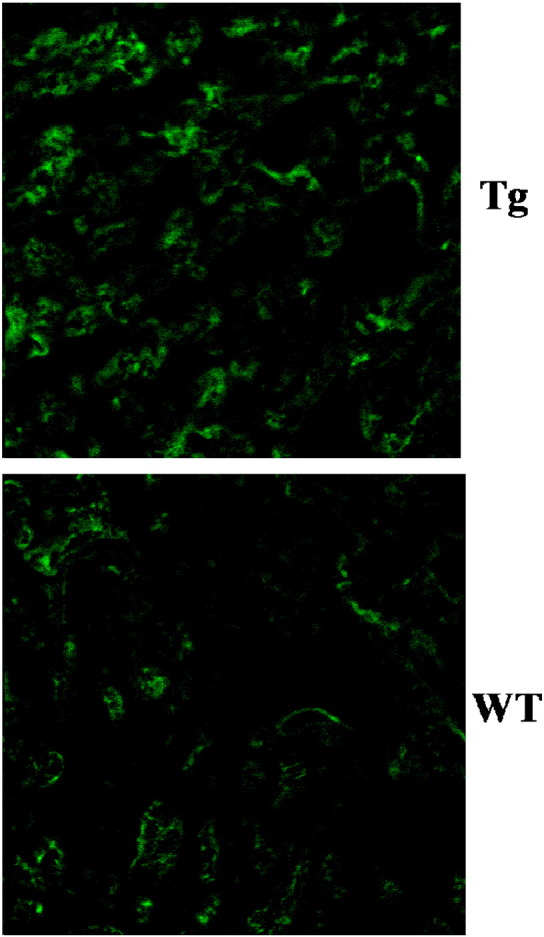
Increased cardiac protein S-nitrosylation levels in P2X4R Tg animals. A typical example of S-nitrosylated proteins in P2X4R-overexpressing Tg and WT hearts (n = 5 for each) is shown as determined using biotin switch method. The counts of total summed intensities produced one distribution for each heart and analyzed with Kolmogorov–Smirnov test for equality of distributions. The test is rejected (P < 0.001) in favor of the distributions not being the same.

We next investigated whether this physical association of P2X4R with eNOS can lead to formation of molecules downstream of eNOS following stimulation of cardiac P2X receptors. Previous results, using the more traditional 4-amino-5-methylamino-2′,7′-difluorofluorescein (DAF-FM) diacetate dye for imaging nitric oxide (NO) in P2X4R Tg cardiac myocytes, showed enhanced fluorescence over the baseline or vehicle-treated fluorescence following 2-meSATP treatment [12]. DAF-FM has the drawback of fluorescence bleaching. Here we report new data with a novel NO-sensitive fluorescent dye, 2-{4,5-bis[(6-(2-ethoxy-2-oxoethoxy)-2-methylquinolin-8-ylamino)methyl]-6-hydoxy-3-oxo-3H-xanthen-9-yl}benzoic acid (FL2E) in its copper complex form (Cu_2_(FL2E)), to image NO formation in biological systems including intact living cardiac myocytes [13], [14], [15]. Our new data, summarized in Fig. 2, confirmed NO formation following 2-meSATP stimulation in the P2X4R Tg myocytes. To determine the physiological relevance of the P2X4–eNOS coupling, we found that WT myocytes also exhibit increased NO formation after 2-meSATP stimulation (Fig. 2b). These data directly demonstrated, using a novel NO-sensitive fluorescent dye, that NO is increased within the cardiac myocytes as a result of P2XR stimulation in not only the P2X4R-overexpressing Tg myocytes but also the WT cardiac myocytes. Thus, using two structurally different dyes specific for NO, we have shown an increased NO level in cardiac myocytes as a result of P2X4R stimulation. NO could cause further downstream activity, most typically stimulation of soluble guanylyl cyclase activation and 3′,5′-cyclic GMP (cGMP) formation, as well as the more recently discovered S-nitrosylation of cellular proteins. Data summarized in Fig. 3 showed that indeed there is an increased level of myocardial S-nitrosylation in the P2X4R Tg hearts compared to WT hearts. The cGMP level is also higher in Tg hearts than in WT hearts, consistent with stimulation of the overexpressed cardiac P2X4R by endogenous extracellular ATP leading to cGMP formation in the Tg hearts [12]. The increased eNOS activity in the P2X4R Tg heart was not due to an up-regulation of eNOS because eNOS protein levels did not change in Tg [12]. Overall, multiple lines of evidence point to a P2X4R-induced intra-myocyte NO production with downstream functional consequences.

## 5 Role of eNOS in mediating the protective effect of P2X4R in HF

Having established activation of eNOS by P2X4R, we next studied the function of eNOS as a mediator of P2X4R protection. We used a pharmacological inhibitor of eNOS, l-N^5^-(1-iminoethyl)ornithine hydrochloride (l-NIO), and gene ablation of eNOS in P2X4R Tg mice during HF. Cardiac transgenic (Tg) P2X4R overexpression caused a higher cardiac left ventricular fractional shortening (FS) after infarction in the P2X4 Tg mice than non-transgenic WT animals. In these studies, permanent coronary ligation caused post-infarct HF but did not influence the infarct size in mice of various genotypes that are subjected to the ligation. Permanent coronary ligation represents a commonly used ischemic HF model. Daily injection with l-NIO—used to demonstrate eNOS function in vivo [16]—abrogated the improved cardiac performance of P2X4R Tg mice during ischemic HF, as determined by both in vivo echocardiography and in an ex vivo working heart preparation.

In further confirming the role of eNOS in mediating the protective effect of the P2X4R during HF, eNOS KO mice were crossed with P2X4R Tg mice (P2X4R Tg/eNOS KO). KO of eNOS abrogated the protected phenotype conferred by the P2X4R Tg genotype in post-infarction HF. Both the improved + dP/dt and FS in P2X4R Tg mice during HF were lost in P2X4 Tg/eNOS KO mice. To ascertain whether the protective effect of P2X4R–eNOS is applicable to another form of HF, we tested the effects of P2X4R-induced protection by pharmacological inhibition of eNOS on pressure overload HF. Pressure overload was produced by aortic banding in P2X4R Tg and WT mice. P2X4R Tg hearts showed better cardiac function as determined by echocardiography-derived FS after aortic banding. l-NIO blocked the increased FS in these animals [12]. KO of eNOS in Tg animals also abrogated the improved FS in these Tg mice after aortic banding. The data provided functional evidence for a P2X4R–eNOS interaction and supported a key role of eNOS as a mediator of cardiac P2X4R-induced protection in HF.

## 6 Protective role of endogenous cardiac myocyte P2X4R in heart failure

While studies in Tg mice with cardiac P2X4R overexpression suggest that cardiac P2X4R is important, the high level (> 20 fold) of receptor expression makes these mice unsuitable to address the physiological role of P2X4R. No direct evidence points to a role for the receptor in health or disease. To study the heart function of the endogenous cardiac myocyte P2X4R, we created a tamoxifen-responsive Cre-mediated knockout (KO) of the P2X4R in cardiac myocytes.

**Fig. 4:**
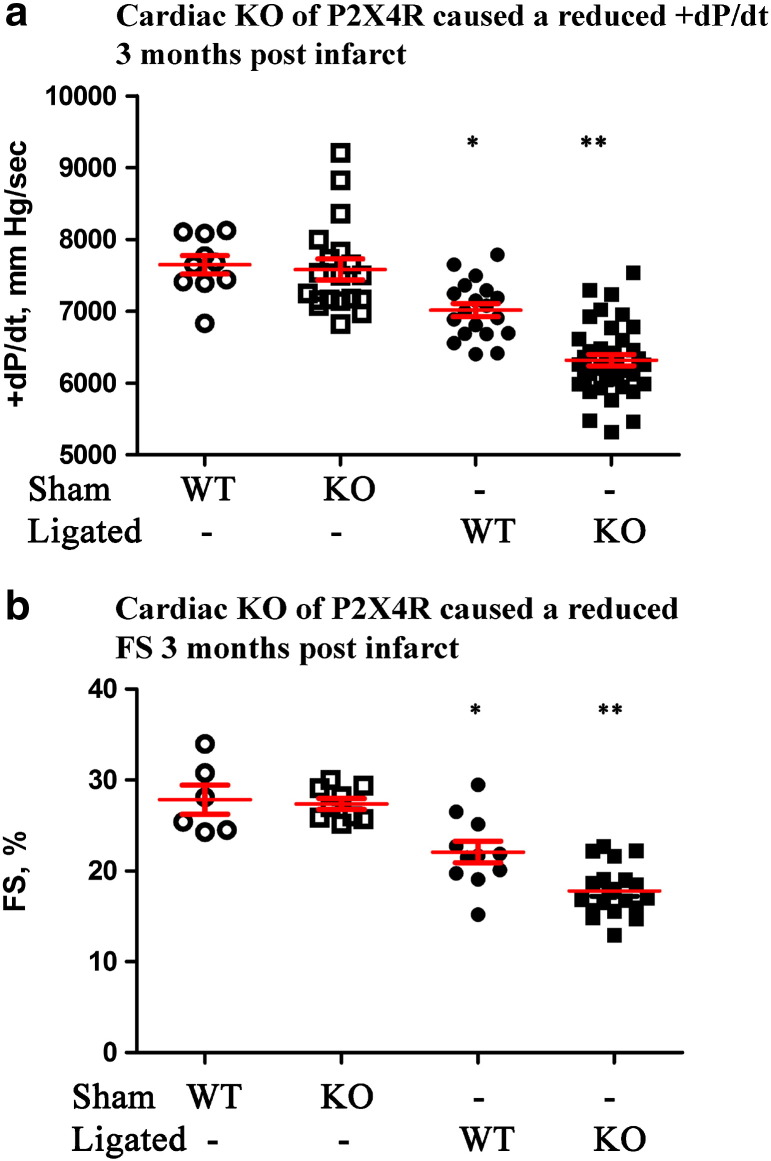
P2X4R KO mice showed a more severe HF phenotype after infarction. WT and KO mice were subjected to sham operation or LAD ligation and cardiac functions determined ninety days later. KO hearts had a lower + dP/dt (a), by ex vivo working heart preparation (n = 39) as well as a more reduced FS by in vivo echocardiography (b, n = 20) than WT hearts (n = 19 and 11 for ex and in vivo measurements respectively). Both WT and KO hearts showed lower + dP/dt and FS than either sham WT (n = 10 for + dP/dt, n = 6 for FS) or sham KO (n = 19 for + dP/dt, n = 9 for FS) hearts. *P < 0.05 for ligated WT vs. sham WT, sham KO or ligated KO. **P < 0.05 for ligated KO vs. sham WT or sham KO. P > 0.05 for sham WT vs. sham KO.

**Fig. 5:**
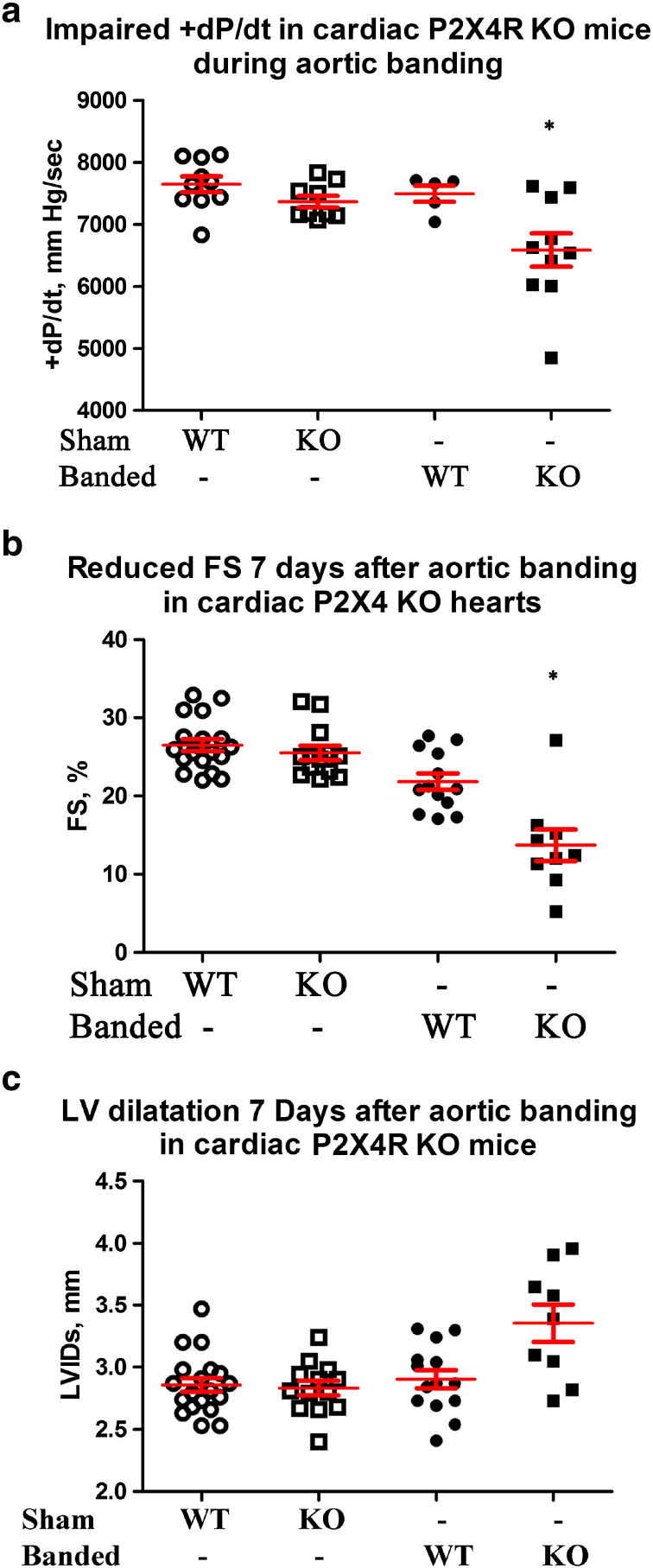
P2X4R KO hearts also had a more impaired function after TAC. WT and KO mice were subjected to sham operation or TAC and cardiac functions determined seven days later. KO mice showed a lower + dP/dt (a) by working heart (n = 10), as well as a more reduced FS (b) and a larger left ventricular internal dimension at systole (LVIDs) (c) by echocardiography (n = 9) than control WT mice (n = 5 for working hearts and n = 13 for echocardiography). KO hearts showed lower + dP/dt, LVdevP and FS than either sham WT (n = 10 for + dP/dt and LVdevP, n = 19 for FS) or sham KO (n = 9 for + dP/dt and LVdevP, n = 13 for FS) hearts. KO hearts also showed more dilated LVIDs than sham WT or sham KO hearts. *P < 0.05 for banded KO vs. banded WT, sham WT or sham KO; P > 0.05 for sham WT vs. KO; P > 0.05 for banded WT vs. sham WT or sham KO in + dP/dt and LVIDs; P < 0.05 banded WT vs. sham WT or sham KO in FS comparison.

Intact cardiac function of P2X4R KO animals was similar to WT control (*P2X4^floxed/floxed^/Myh6* negative) by echocardiography-derived measures and by working heart contractile parameters. KO cardiac ventricular myocytes have the same resting cell length, width and contraction shortening as myocytes of WT littermates. KO hearts were similar to control hearts without any evidence of fibrosis or overt pathology. Thus, cardiac specific P2X4R KO hearts have normal phenotypes without hypertrophy, fibrosis, or cardiac dysfunction at either myocyte or intact heart levels. We then subjected the KO mice to left coronary artery (LAD) ligation or pressure overload by transverse aorta constriction (TAC) or aorta banding. The studies were designed to answer the question on the function of endogenous cardiac myocyte P2X4R under basal conditions or during HF. KO hearts showed a more severe HF phenotype after LAD ligation or aorta banding than WT hearts. Cardiac FS by in vivo echocardiography and + dP/dt by ex vivo working heart evaluation showed impaired heart performance of KO mice in the post-infarct heart failure model (Fig. 4). In the banded animals, KO mice also showed a more decreased cardiac function than WT mice (Fig. 5), indicating a general protective role of the endogenous myocyte receptor in animals with HF caused by different etiologies.

## 7 Summary and outlook

**Fig. 6:**
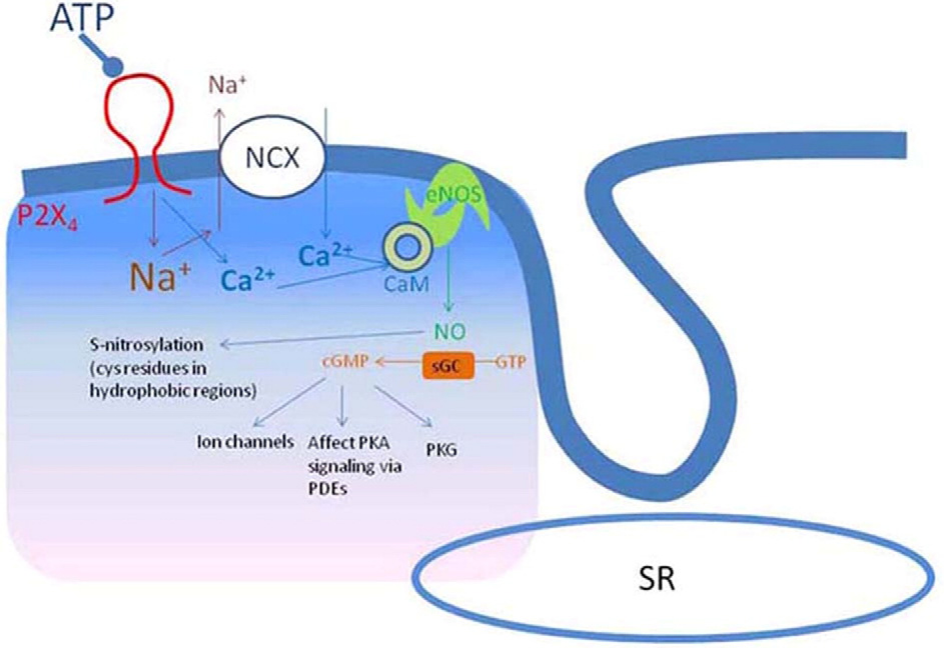
Model of the mechanism of action of P2X receptors in cardiac myocytes. Stimulation of cardiac P2X4R activates eNOS. This is accomplished by a localized increase in calcium near eNOS, binding to local calmodulin and leading to increased eNOS activity. A calcium gradient is set up by diffusion, with minor amounts reaching the SR. NO production can lead to stimulation of two principal downstream effectors, soluble guanylyl cyclase with cyclic GMP accumulation and activation of protein kinase G (PKG) as well as S-nitrosylation of myocardial proteins. These two downstream effectors represent both PKG-dependent and independent pathways, which are known to exert a salutary effect in heart failure and in ischemia/reperfusion injury.

P2X4Rs are ligand-gated ion channels and have emerged as potentially important molecules in regulating cardiac function. The demonstration of a physical P2X4R–eNOS interaction is novel. Although eNOS is cardioprotective, the downstream effectors mediating eNOS activity are unknown (see Fig. 6 for model). Possible candidates include cyclic GMP and PKG or cellular protein nitrosylation [3], [17]. Our data also have implications for the development of a P2X4R antagonist as a new therapy to treat neuropathic pain [18]. Because the endogenous cardiac myocyte P2X4R is cardioprotective, its antagonism during pain control may be deleterious to those individuals under cardiac stress, including HF. Although the role of the P2X4R in other tissues such as the endothelium and monocytes awaits characterization in tissue-specific KO, cardiac-specific activation of eNOS may be more cardioprotective than an increased activity of global systemic eNOS [19]. The intra-myocyte formation of nitric oxide may be more advantageous over nitric oxide derived externally from a donor. A small molecule drug stimulating this sarcolemmal pathway or gene therapy-mediated overexpression of the P2X4R in cardiac myocytes may represent a new therapy for both ischemic and pressure overloaded HF.

## 8 Methods

### 8.1 Mice

The UCHC Animal Care Committee, in compliance with Animal Welfare Assurance, approved all mice handling procedures. P2X4R overexpressing Tg mice were generated as previously described. WT negative littermates and Tg mice were used at 10–12 weeks of age.

### 8.2 Isolation and Measurement of NO Production in Adult Mouse Cardiac Ventricular Myocytes

Ventricular myocytes were obtained from P2X4R Tg or WT mice as described previously [4], [5]. NO formation was measured by epifluorescence imaging of a new cell-trappable NO fluorescent probe FL2E (Strem Chemicals, Inc., Newburyport, MA). The Cu_2_(FL2E) NO dye was prepared and stored according to manufacturer instructions. Before experiments, myocytes were loaded with 5 μM Cu_2_(FL2E) NO dye for 1 h in Tyrode's solution, then plated onto glass bottom dishes for imaging with a Nikon Eclipse TE2000-S. Fluorescence was captured with a 480/40 excitation filter and 535/50 emission filter. Random 40× fields rich in rod shaped striated myocytes were selected and recorded with a CCD camera. Myocytes were incubated with buffer vehicle or 10 μM 2-meSATP (Sigma), and fluorescence was determined at baseline and after 10 min. 100 μM S-nitroso-N-acetyl-d,l-penicillamine (SNAP, Molecular Probes, Life Technologies, Grand Island, NY) was used as a positive control treatment. Fluorescence intensities from individual rod shaped myocytes were quantified using ImageJ (NIH). Twenty to sixty myocytes were counted from each plate before and after treatment for each mouse.

### 8.3 Statistics

Statistical differences were assessed by Student's t test. We considered P values less than 0.05 statistically significant. Standard errors were shown.

## Disclosures

None.

## Figures and Tables

The figure and its legend are reproduced with permission from Wolters Kluwer Health (see  Acknowledgment).

The figure and its legend are reproduced with permission from Wolters Kluwer Health (see Acknowledgment).

The figure and its legend are reproduced with permission from Wolters Kluwer Health (see Acknowledgment).

The figure and its legend are reproduced with permission from Wolters Kluwer Health (see Acknowledgment).

The figure is reproduced with permission from Wolters Kluwer Health (see Acknowledgment).
